# The Road to Personalized Medicine in Alzheimer’s Disease: The Use of Artificial Intelligence

**DOI:** 10.3390/biomedicines10020315

**Published:** 2022-01-29

**Authors:** Anuschka Silva-Spínola, Inês Baldeiras, Joel P. Arrais, Isabel Santana

**Affiliations:** 1 Univ Coimbra, Center for Innovative Biomedicine and Biotechnology, 3004-504 Coimbra, Portugal; ines.baldeiras@sapo.pt (I.B.); isabeljsantana@gmail.com (I.S.); 2 Univ Coimbra, Centre for Informatics and Systems of the University of Coimbra, Department of Informatics Engineering, 3030-290 Coimbra, Portugal; jpa@dei.uc.pt; 3 Univ Coimbra, Faculty of Medicine, 3000-070 Coimbra, Portugal; 4 Neurology Department, Coimbra University Hospital, 3000-075 Coimbra, Portugal

**Keywords:** Alzheimer’s disease, artificial intelligence, AD models, machine learning, data science

## Abstract

Dementia remains an extremely prevalent syndrome among older people and represents a major cause of disability and dependency. Alzheimer’s disease (AD) accounts for the majority of dementia cases and stands as the most common neurodegenerative disease. Since age is the major risk factor for AD, the increase in lifespan not only represents a rise in the prevalence but also adds complexity to the diagnosis. Moreover, the lack of disease-modifying therapies highlights another constraint. A shift from a curative to a preventive approach is imminent and we are moving towards the application of personalized medicine where we can shape the best clinical intervention for an individual patient at a given point. This new step in medicine requires the most recent tools and analysis of enormous amounts of data where the application of artificial intelligence (AI) plays a critical role on the depiction of disease–patient dynamics, crucial in reaching early/optimal diagnosis, monitoring and intervention. Predictive models and algorithms are the key elements in this innovative field. In this review, we present an overview of relevant topics regarding the application of AI in AD, detailing the algorithms and their applications in the fields of drug discovery, and biomarkers.

## 1. Introduction

Alzheimer’s disease (AD) remains the most common neurodegenerative disorder worldwide, with a prevalence of 3.9% for individuals over the age of 60 [1,2]. Being an age-related disease, diagnosis has become a great challenge, and early detection is therefore crucial to allow for future planning and in providing better methods for individuals to be selected for clinical trials before the pathology reaches levels of irreversible neurological loss [3].

AD is characterized by the accumulation of amyloid-beta (Aβ) plaques and tau-related neurofibrillary tangles that affect the prefrontal and mesial-temporal areas of the brain. The detrimental effects of these changes translate to a progressive decline of memory and cognitive function caused by the loss of brain tissue (atrophy) and alterations in neural circuitries [4], especially in those related to acquisition, consolidation, reconsolidation, and extinction of memory and learning [5]. In addition to the neuropathological hallmarks of AD, metabolic [6] and neuroinflammatory [7] pathways are stated to influence the disease course and etiology.

Owing to several failed attempts in delivering effective therapies for AD patients, the general interest has switched from a curative approach to a preventive perspective, where with the intent of testing disease-modifying therapies [8], studies have shifted focus on selecting individuals in preclinical or prodromic stages. In these early phases, patients are largely asymptomatic or present with a pure memory deficit, commonly referred to as mild cognitive impairment (MCI), which could eventually progress to AD-dementia, with a conversion rate of 12–15% per year [9]. Therefore, improving diagnosis and predicting the progression and conversion to dementia constitute major goals in the search towards personalized medicine in AD.

The goal of treatment in AD patients is to mitigate (and whenever possible, improve) cognitive loss and to maintain autonomous function. The pharmacological alternatives for therapy (related to the symptomology) range from targeting the modulation of the affected neural circuitries, by extensive and diverse molecular targets, to the improvement of behavioral manifestations [6]. Alternatively, there are non-pharmacological approaches such as psychotherapy and non-invasive brain stimulation (NIBS). As a novel technique, NIBS has delivered promising results in the restoration of cerebral activity regarding the consolidation and reconsolidation processes [10].

Due to an increased lifespan, the number of patients assisted in dementia clinics has risen and the amount of clinical information continues to grow exponentially, requiring a more technological approach for data analysis. Traditional data analysis methods usually evaluate occurrence, providing results that can lack precision and are time-consuming and costly. Therefore, applying the most advanced techniques can lead to an accurate evaluation of classifications and relationships, that could be generalized to new data. These are suited for handling large and complex data and are typically applied for prediction and pattern recognition [11], and also for complementing a correct diagnosis and categorization into clinical trials. Moreover, storage sustains a crucial role, as the development of databases provide the opportunity to organize, label and classify individuals at a faster rate. Bearing this in mind, there is a need for designing and implementing specific architectures to store the information of interest in a more secure and efficient manner, allowing for easier access and analysis.

Concerning the possibility of improving diagnosis and predicting progression and conversion to AD, it could be beneficial to apply technological approaches such as algorithms, known as artificial intelligence (AI) or machine learning (ML), that are low-cost tools with great performance metrics. In such algorithms, a specific set of features or variables is established, through which a given set of instructions is commanded to discover patterns that can express categorization or associations. Behind this process is a strong mathematical component mainly focused on probability and delineating limits through the measure of distances between points.

The application of precision medicine, with the use of AI techniques, represents an emerging approach for disease treatment and prevention that combines multimodal information with variables such as lifestyle, genetics, physiology, and environmental factors.

This review consists of an overview of the most relevant concepts of the use of AI—highlighting ML and DL models—in AD research. The structure will be divided into two sections: (1) implemented algorithms and their fundaments—omitting the deep technical aspects; and (2) the data-driven applications.

The topics depicted within each section will be selected based on those aspects that were more commonly applied to AD, according to the scientific literature published in the last five years. This review represents an overview of the main characteristics, current applicability, and future perspectives of the implementation of AI in healthcare, specifically in dementia.

## 2. Approaches for Developing ML Models in AD Research

**Figure 1:**
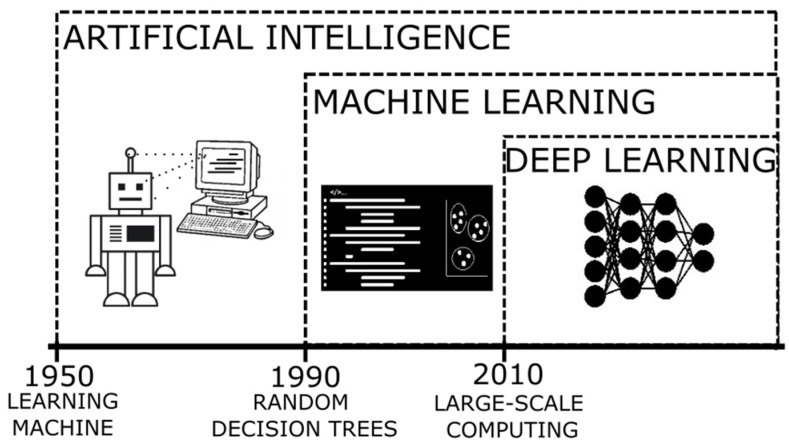
Timeline of the developments of artificial intelligence as an evolution of innovative algorithms.

The terms AI and ML [12] (their relation can be seen in Figure 1) refer to the method where a computer can simulate the human processes of learning and reasoning, by analyzing information and performing tasks following a logical sequence (through a set of instructions). In an oversimplified way, during these processes, when presented with a new object/information, we are able to extract a number of characteristics similar to those of previously known elements (feature extraction) and those that are distinctive of the new elements (rule-based reasoning), and then name or label it to be quickly recognizable (classification). When applying a more complex system, it is expected that the computer can process enormous amounts of information rapidly and reach solutions to complex problems or follow patterns to generate logical results, with a smaller error rate.

**Figure 2:**
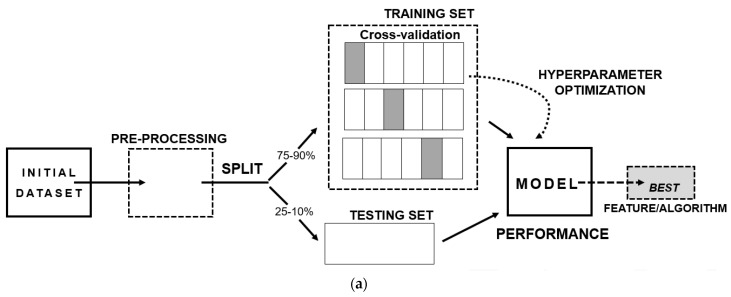

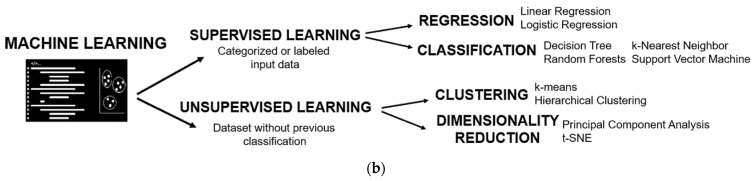
Basis of machine learning: (**a**) Scheme of the stages of development of a machine learning prediction model; (**b**) Types of machine learning algorithms.

The steps to develop an ML model (shown in Figure 2a) consist of three main aspects: (I) A well-structured and well-conceptualized data frame—since the quality of the input data will reflect on the quality of the output-; (II) The separation of the data frame into training and testing data, where it is recommended to add a cross-validation step, as a resampling method, with the intent of excluding results caused by chance; (III) The final model providing the best performance metric, bearing in mind several aspects such as if it answers the scientific question, if it can be generalizable to the population and if the results can be replicated.

Additional to the information presented in the introduction, the advantages of applying algorithms in dementia rely on the capacity of handling data more efficiently and the ability to automate, especially in the development of tools that can aid decision making in the dementia clinics. They also have disadvantages, mainly linked to inadvertent errors and the inclusion of biases. Tackling these limitations is dependent on sound preprocessing methods, where the normalization of the data and the handling of missing values are crucial. As these algorithms are always based on mathematical and statistical principles, they follow the concepts of logic, discrimination, and probability theories.

One of the aspects requiring caution is the possibility of overfitting, a statistical term where the model uses noise as a component negatively influencing the outcome, since it can add the contribution of features that are not part of the underlying distribution. This is the case when there are excessive features or variables in display and insufficient subjects [13]. As a counterpart there is the possibility of underfitting, where there are insufficient variables presented as input.

One key constraint of AI refers to the quality of the data “fed” to the computer. As with any scientific process, the manipulation of the information to be analyzed, where characteristics are fitted to reach a particular purpose, can damage the inference about the underlying pathological process. Hence, the production of biases is generally introduced by the scientist/data analyst, resulting in a compromised output due to erroneous premises. The recommendation of preprocessing to “clean” the data and the removal of certain outliers, translating to an exclusion bias, could tamper the final conclusions. Moreover, with the intent of evaluating generalizability: excessive heterogeneity presented on the sample can harm the results, the same applies to its counterpart (homogeneity). It is always important to keep the research question in mind and the interpretability of the results within the biological context, and to remain aware of the high degree of complexity.

ML algorithms are divided into two branches (Figure 2b): supervised learning, where it is required to present the category or class of the groups of interest, and unsupervised learning, where there is no need to provide a labelled variable of class. Both will be presented and further detailed in the following sections.

### 2.1. Supervised Training

The first scientific article using ML algorithms applied in AD was from Mundt and collaborators in 2000. The objective was to create and evaluate a psychometric screening instrument where the scale and performance metrics would help discriminate between controls and probable Alzheimer’s patients. The authors collected and analyzed several items on surveys performed to caregivers and the neuropsychological scores from patients. They applied a decision tree to discriminate nondemented patients and those with probable AD.

The algorithm used in this work, and decision trees, consist of recursive partitions of data into increasingly homogeneous subsets. Each partition gives rise to a node, that represents a single variable—a decision point—that helps maximize the separation between two groups, in its simpler form as binary classes [14,15]. Although the application could translate to classification, its purpose was to evaluate associations, therefore the statistical process behind it was based on regression.

Since decision trees are susceptible to small changes in the data, altering the structure of the tree and as consequence the results, other algorithms were used for more reliable and robust outputs.

In 2008, Teramoto, published an article based on the application of a semi-supervised algorithm with random forests and a method of label propagation that he concluded was beneficial for having high performance outcomes in predicting an AD patient in the early stages using a small training sample.

Random forests (RF) is a supervised learning method based on decision trees and regression trees. It uses a resampling method creating pseudo-replicates, known as bootstrapping. Each sample creates a tree classifier selecting a variable at each node, and then selects the best split given an averaged result. It is considered a classifier since it constructs the task-specific distance metric, a measure of similarity or ranking [16,17,18].

In the late 2000s, another type of ML algorithm, support vector machine, began taking over in the areas of neuroimaging and genetics. Studies by Capriotti and collaborators [19] in 2006, used this algorithm to predict human neurodegenerative diseases due to a single point mutation at the start of a protein sequence; and Li and collaborators in 2007 [20] used it specifically for processing magnetic resonance images of AD patients with the intent of determining subtle changes in the hippocampus that could discriminate them from controls.

Support vector machine (SVM) is another supervised learning algorithm that provides classification. It is based on a mixture of geometric and probabilistic concepts, in which the goal is to create a separation of two well-defined sections of points, and due to the inherent complexity of the distribution of the dataset, it generates a hyperplane (a mathematical extrapolation to create boundaries in higher dimensions). In the algorithm the selection is determined from the best suited hyperplane, calculated by the maximal distance, and then converted to a separation of clusters in minimal dimensions. One characteristic is that it prevents an unclean separation by allowing some misclassifications, known as a soft margin [21,22,23].

### 2.2. Unsupervised Training

In the early 2000s, unsupervised learning algorithms were also applied in neuroimaging and genetics specifically for AD. Studies by Royall and collaborators [24], and Zhang and colleagues [25], published in 2002, applied hierarchical cluster analysis. The former, sought to determine the contribution of cortical regions of interest in spatial distribution of tauopathy in autopsy material and to perform a dimensionality reduction simply as a statistical method. The latter, used hierarchical cluster analysis to determine gene expression patterns searching for the associated genes for the development of the human hippocampus with those AD-related.

As mentioned above, hierarchical clustering is a statistical method that was implemented as an algorithm. It consists of the identification of distinct groups (clusters), with similar characteristics determined by specific features. It uses a measure of spatial approximation between points in a plane. Additionally, it orders the distances between each possible cluster formation by creating a node, in the shape of a tree, following the maximum likelihood, and generates a dendrogram of this measure [26]. At the end, if there is good separation between the clusters, they would be identified as the classes or categories of subjects.

Besides clustering, another section of unsupervised learning methods consist of those developed to perform dimensionality reductions. Gottfries and collaborators in 2001, published a study where they analyzed multimodal information collected from elderly patients who complained about cognitive disturbances. Of the 19 variables they established, two principal components indicated the two routes of cognitive impairment, one related to one-carbon metabolism and the other related to dementia. They emphasized that the results of the application of principal component analysis should be used to generate a biological hypothesis and explained them as speculations over its generalizability, as a tool that requires further discernment.

Since the data used in health sciences are of increased complexity, having a great number of variable inputs, an enormous effort is required to process them simultaneously. Principal component analysis (PCA) was developed as a tool to reduce the number of dimensions. It determines the lesser components containing most of the data variation, requiring extraction of those that represent more than 90% of variance. It can help to define differences and similarities in the sample. Mathematically, it can be used as a symmetrical covariance matrix, by a specific set of vectors of a linear transformation. Overall, the more-varied component is stated as the most important contributor [27,28,29].

All the algorithms described above, of supervised and unsupervised learning, bear some limitations that have allowed for the deployment of new strategies and new codes. Related to trees and forests, Gradient Boosting (GB) (for example, XGBoost) aimed to minimize errors in the pruning of trees that misclassified the most and allowed for an improved generalization [30,31]. Unsupervised learning has evolved to a greater extent given its plasticity, especially due the advances in neural networks.

One of the technological milestones of recent decades refers to the creation of supercomputers. Due to the improvements in processing capacity, less time is needed for the computer to perform an increasing number of operations, and we now have the power to analyze enormous quantities of information quicker. This has opened the window for the rise of deep learning, also known as artificial neural networks [31].

### 2.3. Deep Learning

Deep learning remains essential in the field of image and language processing, genomics, and drug discovery. Neural networks are based on a specific set of decision rules, in imaging for example, these help to recognize and distinguish between an object and its background. The model is set as a multilayer architecture, based on a series of stages or modules. On each layer the weighted sum of inputs from the previous one results in a non-linear function that passes into the next, referred to as backpropagation, and overall assembling a hierarchical composition of similar features [32]. The layers allow for the amplification of aspects that are established as important in the data, analyzing them in detail at each level, due to the application of several subsampling methods. To perform optimally, it is important to contain a great magnitude of information as an input, hence the concept of big data remains deeply associated to deep learning implementation.

Having covered all the key aspects -in a simple perspective- of ML algorithms applied to AD, we wish to revisit a topic described in the introduction: the databases. Fostering the enrichment of the information collected and analyzed in single clinics to bring in the opportunity of convergence to multicenter studies, which has increased substantially in the number of subjects and provided diversity, thus allowing us to unravel hypotheses about the underlying aspects of the disease from a broader perspective.

Multicenter associations originated with the objective of validating diagnostic tools (such as neuropsychological tests and biomarkers), and continually seek to innovative effective therapies. In the early 1990s, the Alzheimer’s Disease Cooperative Study (ADCS) was created as a consortium of research facilities around the United States of America and Canada fostering new drugs for AD [33]. Otherwise in Europe, the efforts to build co-joint studies came with the creation of the European Union, giving rise to programs such as the EU Joint Programme–Neurodegenerative Disease Research (JPND) and the European Alzheimer’s Disease Consortium (EADC).

Acknowledging the benefits of a multicentric approach led to the foundation and growth of the Alzheimer’s Disease Neuroimaging Initiative (ADNI) database. ADNI started in 2004 in North America with the purpose of validating biomarkers and designing therapeutic trials in AD. This cohort includes subjects with AD, amnestic MCI, and cognitively normal elders. It has enrolled thousands of patients and complies information on clinical, genetic, biofluids, neuroimaging and neuropsychological data that are updated in periods up to 48 months [34]. ADNI remains, up until now, the largest public longitudinal database for AD patients with easy and fast access to information [11].

ADNI remains a major contributor to the progress on the application of ML due to its policies of public access. It has allowed for close to 2000 publications and nearly 300 scientific articles related to AI. It is believed that to further improve this field, it is necessary to start sharing the validated models and apply them in single-center data (protecting the subject’s confidentiality) at a global scale. Moreover, the harmonization of the data collected in dementia clinics and the protocols established in its processing need to be of consensus.

## 3. Main Applications of AI in AD Research

The potentialities of applying AI are vast but limited by data availability (i.e., sample size and data acquisition) and the barriers in the fields of mathematics and physics when considering modelling complex biological systems, where some elements cannot be explained and consequently remain elusive.

In the past 20 years, the field of AD has allowed for the application of new technologies in different areas.

Having access to big data, the fields of neuroimaging and genetics [35,36] are more prone to work with algorithms. Moreover, with the creation of biobanks and telehealth, the field of fluid biomarkers expanded its studies to a more multimodal approach, integrating large amounts of clinical and neuropsychological information for the development of models.

**Figure 3:**
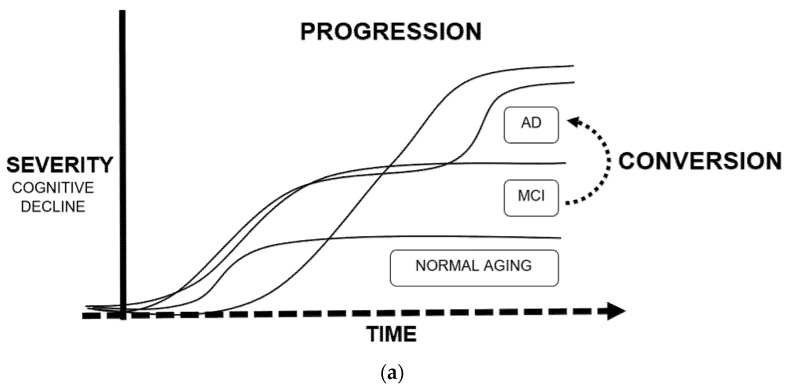

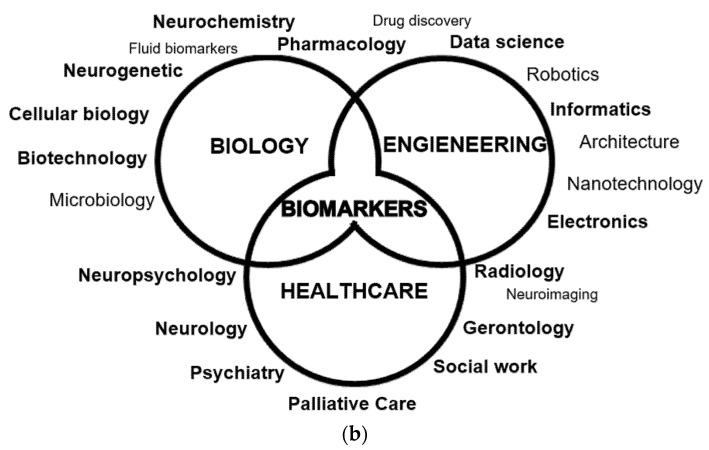
Main applications of machine learning in Alzheimer’s disease: (**a**) Definition of progression and conversion as time-to-event measures due to cognitive decline; (**b**) Fields of study of Alzheimer’s disease, converging in the development of biomarkers.

Tackling the theme of the applications of ML in AD in greater detail, we will dedicate this subsection to the five more relevant and abundant topics on scientific publications: drug discovery, neuroimaging, biomarkers, conversion, and progression (exemplified in Figure 3).

### 3.1. Neuroimaging

As mentioned before, one of the major advancements performed with ML and deep learning algorithms was in image processing. Since the discovery of X-rays in the 19th century, and the development of computerized tomography (CT), medical imaging/radiology has been a discipline intertwined with technological and scientific breakthroughs. Seventy-six years later arose the invention of magnetic resonance imaging (MRI) and, a few years later, positron emission tomography (PET). All of these techniques allowed researchers to visualize and measure the physiology of an individual and since their discovery there has been a continuum of innovative enhancements to improve the precision of these images.

Neuroimaging represents a crucial tool on the characterization of AD. MRI images allow for the evaluation of the structural status of the brain and are usually used to establish neurological damage or atrophy [15,37,38,39,40,41,42]. Additionally, functional MRI [23,29,43,44] can be performed for detecting brain activity through the changes in blood flow. PET scans [39,40,45,46,47,48] are more invasive, expensive, and require the administration of specific radioactive tracers that allow for the measure of specific changes in metabolic and physiological processes.

Tam et al. (2019) developed a multimodal signature of Alzheimer’s dementia from T1-weighted MRI scans from cognitively normal (CN), MCI and AD patients from the ADNI database. They applied voxel-based morphometry analysis, with segmentation into probabilistic maps, normalized to a predefined grey matter template and smoothed by a Gaussian blurring kernel. They merged these results with demographic, clinical, neuropsychological, and cerebrospinal fluid (CSF) information and developed a SVM prediction model with an accuracy of 0.949 differentiating between CN and AD. They reached the conclusion that in MCI patients, the cognitive features have a higher predictive score than structural ones and that there were some redundancies between cognition and atrophy.

Moreover, Nguyen et al. (2019) used the ADNI database to extract information from CN, MCI, and AD patients, and used for comparison a cohort from the Chosun University Hospital. In this case, they acquired the measures from resting-state functional MRI (rs-fMRI). They performed several steps of preprocessing to guarantee good quality imaging (calibration, realignment, normalization, smoothing, etc.); the structural images (T1-weighted) were co-registered to the functional ones after realignment. They developed a SVM model, after a leave-one-out cross-validation and dimensionality reduction obtaining an accuracy of 0.988. Lastly, they extracted the regions of interest (ROI) with significant changes, determined by a univariate t-test, showing a pattern of discrimination in the prefrontal cortex and cingulate cortex/precuneus. Additionally, they obtained different significant regional features according to the dataset, hinting at demographic differences.

Regarding studies with PET, three types of PET methodologies are performed according to the target of the radiotracers: metabolic PET, usually performed with fludeoxyglucose (FDG) [15] details the metabolic activity in the brain, as a measure of connectivity; due to the rapid intake of glucose any pathological effect such as neuronal death or the formation of plaques, can decrease the signal. Amyloid PET, usually performed with Pittsburgh compound B (PiB) and Florbetapir, displays affinity for amyloid deposits and is used for Alzheimer’s and cerebral amyloid angiopathy (CAA) studies. In addition, tau PET with Flortaucipir, allows for the estimation of the aggregates of neurofibrillary tangles mainly composed of phosphorylated tau protein. PET studies have also been the subject of ML approaches, as described in the following examples:

Ding et al. (2019) developed a deep learning model using FDG-PET for early prediction of AD. They used longitudinal data of ADNI database and also tested the model on a small cohort (*n* = 40) from patients referred to Californian memory clinics. Similar to the studies on MRI, they followed several steps of preprocessing. They created a convolutional neural network, based on 14 million images of 1000 classes from the ADNI dataset. The model resulted in an AUC for prediction of AD, MCI, and non-AD/MCI of 0.92, 0.63, and 0.73, respectively.

Ezzati et al. (2020) created a predictive ML model for Aβ+ risk. They extracted multimodal information (neuropsychological scores, MRI and PET scans, ApoE4 status, CSF biomarkers, and demographic information) from amnestic MCI patients of the ADNI database. They labelled the patients as Aβ+ and Aβ− according to the result from the processing of the Florbetapir PET images. The algorithm used was Ensemble Linear Discriminant, a classification method that results in the higher accuracy from an ensemble decision rule obtained from each individual classifier and was optimized by selecting the best hyperparameter. After evaluating several feature combinations as models, the best one contained demographics, ApoE4, and CSF markers given an AUC of 0.86.

Jo et al. (2020) established a 3D convolutional neural network using the Flortaucipir PET scans from CN, MCI, and AD patients of the ADNI database. Additionally, they applied a layer-wise relevance propagation algorithm to determine the contribution of a single pixel (input) to the prediction of the classification task (output), a more detailed measure of accuracy. Based on the model, they calculated the AD probability scores of the MCI patients, separated into early (EMCI) and late (LMCI). The greater AD probability score was associated with increased accumulation of tau in the medial temporal lobe, with a correlation of 0.49 for LMCI. Finally, they concluded that the classifier associated the tau deposition in LMCI to be more similar to AD than the EMCI participants.

As described by these studies, the use of multimodal imaging data, where the authors were able to extract information about structural and functional abnormalities [16], increased the discriminatory capability, mainly when there was an overlapping presentation. One of those cases was exemplified by a “mixed” vascular dementia (VD)–AD profile, where the progressive cognitive impairment resulted from brain tissue damage caused by vascular disease which could be misinterpreted as AD-related. As a result, the use of diffusion tensor imaging (DTI) from MRI studies can help elucidate the distinct patterns of white matter changes determining a distinction between VD and AD [4].

### 3.2. Multimodal Biomarker-Based Studies

The framework of AD diagnosis and research has changed dramatically through the development and application of biomarkers. Core AD biomarkers include: (1) cerebrospinal fluid (CSF) low levels of Aβ42 or the Aβ42/40 ratio and brain amyloid deposition evidenced by PET imaging; (2) increased total tau (t-Tau) and phosphorylated tau (p-Tau) representing cortical neuronal loss and cortical tangle formation, respectively; and (3) hippocampal atrophy shown on MRI [34]. These markers showed high diagnostic accuracy for established AD [42,49] and identified AD before onset of dementia at the MCI stage, evidenced in single center [1,50] and large-scale multicenter studies [51]. Their high sensitivity and specificity –between 85–95% if combined–led to their incorporation in diagnostic criteria, proposed by the National Institute of Aging-Alzheimer Association (NIA-AA) for AD dementia [34], MCI [52] and preclinical states [53].

However, given the constraints related to neuroimaging and the collection of CSF samples (respectively, cost and invasiveness), efforts were put into the development of more sensitive instruments and methods to be able to quantify brain-derived proteins through immunoassays in blood [54]. Nowadays, blood measures of the core AD biomarkers were proven effective and showed promising results in several studies [55,56,57,58].

Although single biomarker studies usually perform well as indicators of disease studies, using ML models, that gather as much variables as possible, and allows for the development of multimodal biomarker studies, to try to discern patterns that due to its global complexity could be overlooked at first sight. An example of such an approach was seen in the study of Beltrán and collaborators (2020) who focused on blood biomarkers. They developed a predictive model of Alzheimer’s progression based on a combination of plasma biomarkers, due to the variety of potentially involved pathways. The model was based on the ADNI database, and they selected the best model out of several ML algorithms implemented (classification and regression tree, GB, RF, and SVM). Additionally, a PCA and a feature reduction analysis was performed. The authors created two classes of binary outcomes (stable and progressive) taking into consideration the interval of transition from MCI to AD. The plasma biomarkers were divided accordingly by groups as cardiac, inflammatory, metabolic, and neuronal markers. Finally, RF and GB exhibited the highest AUC. Overall, they highlighted the potential applicability of blood biomarkers as a first contact to determine risk and transition to additional testing for confirmation (as MRI, PET or CSF sampling).

The potential capability of the application of blood biomarkers is the differential diagnosis between neurodegenerative dementias. Lin et al. (2020) created a linear discriminant analysis model with a RF classifier based on plasma biomarkers (Aβ42, Aβ40, t-Tau, p-Tau181, and α-synuclein) from healthy controls, patients with AD spectrum, Parkinson’s disease (PD) spectrum, and frontotemporal dementia (FTD). The model displayed an accuracy of 0.76 when classifying the neurodegenerative disorders and was capable of distinguishing disease severity on the AD and PD spectrums with an accuracy of 0.83 and 0.63, respectively.

Another important component of biomarkers studies is a neuropsychological assessment [59]. One key component of the generation of classes for supervised learning models are based on the score obtained by the Clinical Dementia Rating (CDR). This is a staging tool crucial to determine whether an individual is diagnosed as MCI or AD, and is usually complemented by the Mini Mental State Examination (MMSE) score, which measures overall cognitive status. For instance, the study by Yao and colleagues (2020 [60]. described the use of a ML model (GB and SVM) that helped to generate a cognitive resilience score, which they defined as the difference between the observed and expected cognitive status displayed by an AD patient with a specific presumed level of AD pathology. They used the data from two longitudinal cohort studies (the Religious Orders Study and the Rush Memory and Aging Project) and the information from a battery of 21 cognitive tests administered annually, that assessed five cognitive domains (episodic memory, semantic memory, working memory, perceptual speed, and visuospatial ability). Additionally, information regarding comorbidities, demographics, lifestyle, and postmortem neuropathological evaluation was included. The predictive performance with measures collected at baseline reached an accuracy of 0.77 and was suggestive that the model could be applied as a tool for intervention in subjects that were classified as having low cognitive reserve.

Another promising source of biomarkers in AD is neuro-ophthalmological evaluation. The retina is often referred as the window into the brain since the morphological changes in the brain during the neurodegenerative process are replicated in the retina. In the study by Nunes et al. (2019) optical coherence tomography was used to assess the thickness of inner retinal layers performing a texture analysis in healthy controls, AD and PD patients. They analyzed the data collected and applied SVM as a classification model, with an accuracy up to 0.88. This method represents a simple, inexpensive, and non-invasive tool for early diagnosis that could be implemented for assessing neurodegeneration in addition to other techniques.

### 3.3. Conversion and Progression

Regarding the application of ML algorithms in AD using longitudinal data, the most frequent objective was the development of prediction models that determine the risk/time of conversion from MCI to AD [22,30,61,62,63,64,65,66,67,68,69], or the course of the disease in terms of severity [50,70,71,72,73,74,75,76,77,78,79,80]. These models are based on time, which adds another layer of complexity. Repeated measures design, as a statistical method, is well-established and helps to validate results while maintaining low variability, yet time-to-event prediction in ML remains more challenging.

Longitudinal studies that are data-driven bear several limitations, having to maintain consistency for long periods of time usually requires more investment of resources and a higher risk of dropouts. Additionally, among the AD spectrum the progression of the disease is, in its majority, extremely slow taking up to decades. Embarking on a project that requires decades, to provide astonishing results, and preserving thousands of patients with regular visitations, would be regarded as a titanic endeavor.

Taking into consideration these issues, technological approaches were developed towards digitalization. For instance, the collection of data could be performed remotely through tracking devices [81,82], telemedicine consultations [83] or an array of sensors and gadgets installed at home [84]. Although these may seem expensive at first, it is expected to decrease the indirect costs that accumulate with time.

Without the implementation of these technologies, another approach has been the development of models that can predict dropouts and failed appearances to scheduled medical appointments [85], and application of different techniques of data imputation [75] or creation of simulations [37] with the objective of substituting missing values and somewhat improving the quality of the data.

Grassi et al. (2019) reported that only a fraction of 20 to 40% of MCI patients progress to AD within 3 years after the initial diagnosis. Therefore, they used the information from the ADNI database from 550 MCI patients classified as converters and non-converters with at least 3 years follow-up and applied 13 supervised ML algorithms based on a weighted rank average ensemble of them all reaching an AUC of 0.88, that ultimately would determine the probability of an individual with a specific set of values of being a converter during that 3 year period. To determine an exact timepoint of conversion, Khanna and collaborators (2018) also used the information available in the ADNI database over 8 years of study and generated a GB algorithm with a Kaplan–Meier estimator generating a time-to-event output with an AUC of 0.86. Another approach presented by Moscoso et al. (2019) was to study the MRI changes of stable MCI patients, also fetched from the ADNI database, through stratified intervals of time (2 and 5 years) and obtained a signature of volumetric measures for converters.

Regarding progression, Bhagwat et al. (2018) developed a model for prototypical symptom trajectory based on the multimodal data from ADNI and the Australian Imaging, Biomarker and Lifestyle Flagship Study of Ageing (AIBL), where they applied hierarchical clustering to obtain nine timepoints through 6 years. For further analysis they generated five trajectory classes: stable and decline, based on MMSE and stable, slow-decline, and fast-decline based on ADAS-Cog 13 scores, through a longitudinal Siamese neural-network with an accuracy of 0.9. In a similar approach, Geifman and collaborators (2018) obtained three distinct subgroups of patients: rapid decliners, slow decliners, and severely impaired slow decliners through the application of latent class mixed algorithm based on datasets from 18 studies. Moreover, Fisher et al. (2019) applied an unsupervised learning algorithm, Conditional Restricted Boltzman Machine—a method that generates imputations of missing data-, to simulate in detail the patients’ trajectories based on the Coalition Against Major Diseases (CAMD) Online Data Repository for AD (CODR-AD) as a forecasting tool.

Due to the variability in clinical manifestations and route of progression exhibited in the AD patients, it was suggested that the diagnosis of probable AD should be stratified in subtypes [86]. The examples described above attempted to resolve this premise, dealing with the increased complexity through the application of more intricate unsupervised learning algorithms and assessment of simulations. It is expected that this synthetic approach would be replicated in real conditions, although at this point, they still require validation.

### 3.4. Drug Discovery

Traditional methods of drug discovery and development are expensive, time-consuming, and represent a high risk. The advancements in AI allowed for the development of technological-oriented methodologies. These in silico analyses allowed for the assessment of drug design [17,87,88,89], repositioning [90,91], and pharmacological combinations [92,93]. Additionally, contributed as tools used in genetic [94,95] and immune-targeted [7,96] therapies.

As an example of drug design, Vignaux and collaborators published a study in 2020 where they developed a Bayesian ML model based on the CHEMBL and PubChem openly available data for AD-related proteins, with the intent of finding a novel small-molecule that could be administered as treatment. They selected glycogen synthase kinase 3 beta (GSK3β), an enzyme that phosphorylates tau protein, as a target of interest. Ultimately, the model identified several small-molecule inhibitors already approved for safety, extracted from the SuperDRUG2 library. The model for GSK3β used a 732.8 nM threshold analyzed 2368 compounds and reached a precision of 0.858 after a 5-fold cross validation. Afterwards, they evaluated the GSK3β IC_50_ prediction model to score the SuperDRUG2 database for a measure of the confidence in the inhibitory activity of compounds against the target and its applicability, finally selecting ruboxistaurin (prediction score = 0.76, applicability = 0.82). Then, they tested in vitro the top five best performing inhibitors, with ruboxistaurin displaying the highest inhibition (96% at 100 µM).

On in silico drug repurposing, Zeng et al. (2019) developed a deep learning model (deepDR) based on a multimodal deep autoencoder with the objective of systematically inferring new drug-disease associations. They assembled clinical and experimental validated drug-disease network data from DrugBank and repoDB. Then, they integrated the high-level features into multiple networks and constructed a low-dimensional feature representation. The model predicted drug-disease relationships validated by ClinicalTrials.gov database with an area under the curve (AUC) of 0.83 and identified 20 candidates for AD.

Anastacio (2019) estimated the correlation of the epidemiological benefit of drug combinations, derived from the Rush Alzheimer’s Disease Center (RADC) database. The computational model mimicked the microglia-mediated neuroinflammation by inputting the cell activation of receptors, cell-signaling pathways, and protein expression. The model was a recurrent network learning algorithm of nonlinear units bounded by a binary range that received 90 inputs of endogenous and exogenous receptor ligands and drugs. The parameters were the weights of the connections between the elements and were optimized by the results of in vivo and in vitro experiments described in the literature. He concluded that the ten best drug combinations included at least two of the main type of drugs used to treat hypertension, and the use of aspirin with the already established medication approximate (in efficacy) to the results of the antihypertensives.

## 4. Discussion

The applications of ML in AD for the fields of neuroimaging, biomarkers, conversion, progression, and drug discovery, supported the implementation of complementary tools for disease prevention, diagnosis, patient monitoring, and development of new protocols for treatment, and mainly contributed to the process of analyzing voluminous data in an accurate and efficient manner moving towards automation.

From a medical standpoint it remains important to highlight, that although there are clinical guidelines for diagnosis of probable AD, there remain challenges in providing a correct diagnosis [34,55]. Moreover, there are early-and late-life onset, presymptomatic and symptomatic stages, and typical and atypical clinical manifestations, and as a result AD is defined as a spectrum [6]. Since definite diagnosis can only be acquired postmortem, there are many unknowns standing from disease onset to the course that each patient follows. Predicting how an individual will evolve (at which rate and how severe it would be), represents one of the main goals for the use of ML tools.

Concerning radiology, due to the high costs associated with equipment, structural and functional neuroimaging continues to mainly evolve in improving the quality of the images and refining the software for processing and analysis (for example, more specialized and diverse Atlases—the topographic collection of brain images delimited and labeled-). In ideal conditions, AD patients should be evaluated at baseline with MRI and PET scans and continue these procedures alongside follow-ups, but the resources (monetary, infrastructural and of staff) are substantial and cannot be supported by every health facility, therefore neuroimaging is complemented with other measures.

As seen in the previous section, the strength of the uses of multimodal information allows for the advancements in AD research proceeding towards personalized medicine, developing a set of precise categories (as code) that estimates the changes during the disease course of each patient (developing a signature or profile), and for the addition of validated and accurate biomarkers, such as the novel and automated blood based measures.

Computational tools are supporting the change towards improvements for precise, fast, cost-effective, and non-invasive strategies for correct diagnosis and monitorization, from CSF to blood-based biomarkers to the use of less harmful neuroimaging techniques such as DTI and novel PET radiotracers.

Moreover, the application of data mining allows researchers to comprehend the depth of details in the classification process, combining the analysis of multifactorial information and time, which would be useful in customizing treatment when the appropriate therapies become available.

Regarding the treatment for AD, efforts sustained into achieving successful therapies had given mostly negative results. Pharmacological companies have spent enormous amounts of time and money into the field of dementia resulting in failed attempts. The reason behind those negative results puzzles experts to this day; it is believed that there are many unknown factors surrounding the underlying neuropathological causes of AD and/or that the participants on the clinical trials are already at a phase of deep neurological loss that cannot be reverted, and the progression could be too small or even eclipsed by other detrimental effects.

The lack of efficient treatment disengages the relationship between diagnosis and therapeutics, where the clinician is left with a surrogate strategy: the medication prescribed targets the symptomatology, alleviating some of the detrimental effects associated with cognitive deterioration [6], as mentioned in the introduction.

Even with the promising results of aducanumab [97] and its conditional approval by the United States Food and Drug Administration (FDA) as a treatment for AD in June 2021, skepticism remains among clinicians. Other Aβ plaque-targeted antibody drugs such as gantenerumab and solanezumab proved inefficient on patients with dominantly inherited Alzheimer’s disease (DIAD) [98]. These controversial results in several clinical trials prove that it is necessary to continue funding studies for drug discovery in AD complemented with computational tools.

These in silico approaches require a multidisciplinary structure of research. They represent the first step in the discovery of new compounds that are theoretically efficient. The next stage should consist of preclinical studies directed to test these drugs and ultimately, to administer them in clinical trials.

In general, the application of ML algorithms in AD represents an emerging field that is moving fast. The technological advancements in healthcare consolidate the future of our society in the coming years. Even with some degree of resilience, these methods represent a tool to enhance the skills of the health professionals, and overall, the objectives rely on increasing the depth of knowledge and providing better care for affected patients.

Lastly, the AI-specialized teams continue to address these concerns and persist in trying to create solutions seeded from academic, business, and governmental institutions.

## 5. Limitations and Future Directions

From a technical standpoint, there were some weakness presented in these studies that are worth highlighting. The nature of the data presents an inherent limitation, since the authors worked with a convenience sample, where the data was obtained from dementia clinics or by accessing databases, the sample size was insufficient (underpowered) when working with a high number of variables, returning to the matter of overfitting (already described), where something could emerge as significant but could simply be a product of chance [99].

Additionally, these methods of data acquisition can lead to selection bias where the researcher might be moving away from reaching a representative profile of the population and inadvertently increasing the sampling error, which leads to a misspecified model.

It is not necessary to accumulate enormous amounts of information to answer a scientific question in statistical ML but remains preferable to gather pertinent and sufficiently sparse data that is representative of the population. On the other hand, missing data and outliers are sources of useful information and are increasingly difficult to track when big data is used.

After seeing all of these studies performed with AI algorithms one can only questions what is the best algorithm or why there are so many and what do the outputs mean? At this moment, we can only define this work as a beta-test. These models require to further testing and validation in real conditions (the clinical context), and time is necessary to see how well they perform. Most of these models need to be inserted into a learning loop where they can evolve with the introduction of new data.

Even with the diverse and expanding amount of literature related to AI techniques and ML models in AD research, the overall assessment suggests that the researchers presented the highest possible measure of how accurate the new data was classified, for values above 80%, and aimed for a balanced and high percentage of sensitivity and specificity (correct classification), that fluctuates between 65 and 98. Having these different results represents a constraint that requires an ongoing process that remains to be refined, especially when the human diagnosis (referred as the one given by the neurologist) remains already pertinent.

In summary, these studies represent an initial stage with some known challenges: the need for standardization of the procedures, the harmonization of the data, translation of these theoretical models to the clinical context, the generalizability of the results and the integration of multidisciplinary teams.

At this moment, the multicentric collaborations, such as that by van Maurik and collaborators (2019) who undertook the comparison of model performance of different European datasets, reaching 2611 individuals with MCI in four cohorts, and obtained only a limited sample size.

Therefore, it may not be enough to apply ML techniques on a few hundred or thousands individuals from a disease that affects millions, and a solution may be to resort to traditional sampling tools and build global networks with multicenter collaborations, guaranteeing data quality and representativeness.

Moreover, there is the need to replicate these studies on independent datasets of appropriate size, that could be more adequate to those high dimensionality problems that researchers are trying to answer, and to being able to corroborate the results obtained.

## 6. Conclusions

This review has introduced the most recent and noteworthy computational advances in AD. We hope that the contents will help to acknowledge the strengths of this area and motivate the creation of projects for preventive therapies and the engagement of multidisciplinary teams. These techniques represent a tool that would only enrich the basic sciences and clinical practices working altogether to reach a mutual goal: to improve healthcare in dementia. Certainly, this field will continue moving forward to help provide solutions for the aging population.
